# 6-{[(Benz­yloxy)carbon­yl]­oxy}-2-methyl­hexa­hydro­pyrano[3,2-*d*][1,3]dioxin-7,8-diyl bis­(chloro­acetate)

**DOI:** 10.1107/S1600536810004356

**Published:** 2010-02-10

**Authors:** Jerry P. Jasinski, Ray J. Butcher, M. T. Swamy, H. S. Yathirajan, B. Narayana

**Affiliations:** a Department of Chemistry, Keene State College, 229 Main Street, Keene, NH 03435-2001, USA; b Department of Chemistry, Howard University, 525 College Street NW, Washington DC 20059, USA; c Department of Studies in Chemistry, University of Mysore, Manasagangotri, Mysore 570006, India; d Department of Studies in Chemistry, University of Mysore, Manasagangotri, Mysore 570 006, India; e Department of Studies in Chemistry, Mangalore University, Mangalagangotri 574 199, India

## Abstract

The asymmetric unit of the title compound, C_20_H_22_O_10_Cl_2_, consists of a 6-{[(benz­yloxy)carbon­yl]­oxy}group and two chloro­acetate groups bonded to a 2-methyl­hexa­hydro­pyrano[3,2-*d*][1,3]dioxin group at the carbon 1,2 and 3 positions, respectively, of a pyrano ring fused to a dioxin ring. The dihedral angle between the mean planes of the dioxin and benzyl rings is 42.2 (2)°. An extensive array of weak inter­molecular C—H⋯O hydrogen bonds links the mol­ecules into chains along [011]. Additional weak inter­molecular C—H⋯π inter­actions occur between C—H atoms of the dioxin and benzyl rings and a nearby benzene ring. A MOPAC geometry optimization calculation *in vacuo* revealed that the dihedral angle between the mean planes of the dioxin and benzyl rings increased by 24.42 to 66.64°, suggesting that the weak inter­molecular hydrogen-bonding inter­actions, in coord­ination with weak C—H⋯π inter­actions, influence the geometry of the resultant crystalline species and help to stabilize the crystal packing.

## Related literature

For background to the title compound, see: Ernst & Derendorf, (1995 ▶); Ji *et al.* (1997 ▶); Sanford *et al.* (1990 ▶); Budavari (1989 ▶); Wrasidlo *et al.* (2002 ▶). For related structures, see: Shi & Wang, (2003 ▶); Wu *et al.* (2005 ▶); Zhou *et al.* (2005 ▶). For bond-length data, see: Allen *et al.* (1987 ▶). For puckering parameters, see: Cremer & Pople (1975 ▶). For MOPAC PM3 calculations, see: Schmidt & Polik, (2007 ▶).
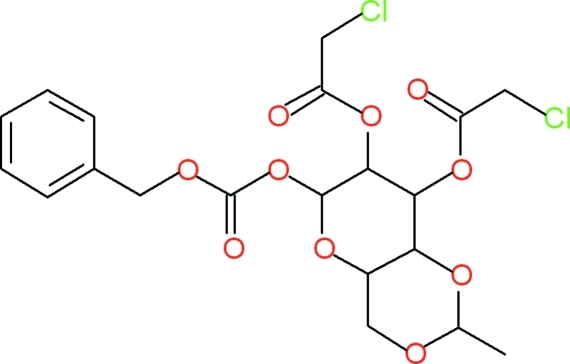


## Experimental

### 

#### Crystal data

C_20_H_22_Cl_2_O_10_
*M* *_r_* = 493.28
Orthorhombic, 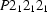

*a* = 8.1780 (1) Å
*b* = 14.9165 (3) Å
*c* = 19.3555 (4) Å
*V* = 2361.12 (7) Å^3^
*Z* = 4
Mo *K*α radiation
μ = 0.33 mm^−1^
*T* = 200 K
0.44 × 0.34 × 0.27 mm

#### Data collection

Oxford Diffraction Gemini diffractometer
Absorption correction: multi-scan (*CrysAlis RED*; Oxford Diffraction, 2007 ▶) *T*_min_ = 0.821, *T*_max_ = 1.000
30676 measured reflections
5818 independent reflections
3677 reflections with *I* > 2σ(*I*)
*R*_int_ = 0.049

#### Refinement

*R*[*F* ^2^ > 2σ(*F* ^2^)] = 0.040
*wR*(*F* ^2^) = 0.084
*S* = 0.92
5818 reflections
290 parameters
H-atom parameters constrained
Δρ_max_ = 0.34 e Å^−3^
Δρ_min_ = −0.23 e Å^−3^
Absolute structure: Flack (1983 ▶), 2513 Friedel pairs
Flack parameter: 0.05 (5)

### 

Data collection: *CrysAlis PRO* (Oxford Diffraction, 2007 ▶); cell refinement: *CrysAlis PRO*; data reduction: *CrysAlis PRO*; program(s) used to solve structure: *SHELXS97* (Sheldrick, 2008 ▶); program(s) used to refine structure: *SHELXL97* (Sheldrick, 2008 ▶); molecular graphics: *SHELXTL* (Sheldrick, 2008 ▶); software used to prepare material for publication: *SHELXTL*.

## Supplementary Material

Crystal structure: contains datablocks I. DOI: 10.1107/S1600536810004356/hg2627sup1.cif

Structure factors: contains datablocks I. DOI: 10.1107/S1600536810004356/hg2627Isup2.hkl

Additional supplementary materials:  crystallographic information; 3D view; checkCIF report

## Figures and Tables

**Table 1:** Hydrogen-bond geometry (Å, °) *Cg*3 is the centroid of the C10—C15 ring.

*D*—H⋯*A*	*D*—H	H⋯*A*	*D*⋯*A*	*D*—H⋯*A*
C6—H6*B*⋯O8^i^	0.99	2.57	3.235 (3)	125
C13—H13*A*⋯O10^ii^	0.95	2.54	3.452 (4)	162
C17—H17*B*⋯O5^iii^	0.99	2.42	3.310 (3)	149
C19—H19*A*⋯O3^iv^	0.99	2.52	3.460 (3)	158
C19—H19*B*⋯O2^v^	0.99	2.38	3.364 (3)	170
C20—H20*B*⋯O4^iv^	0.98	2.59	3.494 (3)	154
C4—H4*A*⋯*Cg*3^iv^	1.00	2.89	3.879 (2)	171
C14—H14*A*⋯*Cg*3^vi^	0.95	2.87	3.818 (4)	173

Symmetry codes: (i) 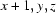; (ii) 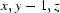; (iii) 
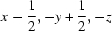
; (iv) 
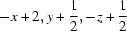
; (v) 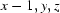; (vi) 
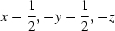
.

**Fig. 1.**
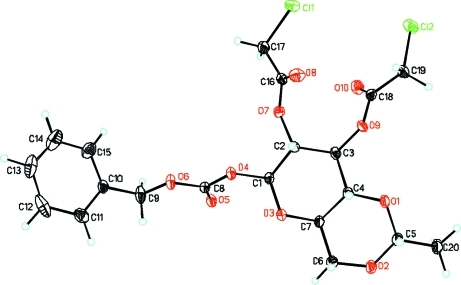
Molecular structure of (I), C20H22O10Cl2, showing the atom labeling scheme and 50% probability displacement ellipsoids.

**Fig. 2.**
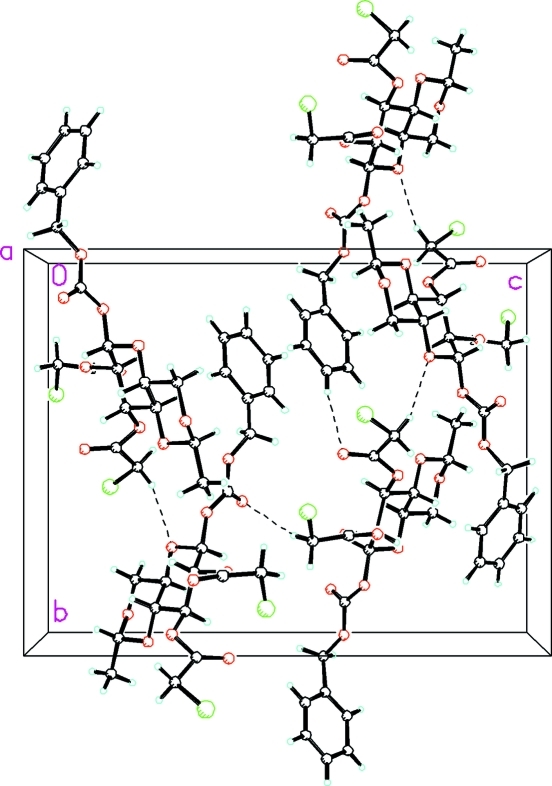
The molecular packing for (I) viewed down the a axis. Dashed lines indicate weak C—H···O intermolecular hydrogen bond interactions which link the molecule into chains propagating along the [011].

## supplementary crystallographic information

### Comment

The title compound is an intermediate in the preparation of etoposide phosphate (Budavari, 1989), an inhibitor of the enzyme topoisomerase II. It is used as a form of chemotherapy for malignancies such as Ewing's sarcoma, lung cancer, testicular cancer, lymphoma, non-lymphocytic leukemia, and glioblastoma multiforme. It is often given in combination with other drugs. Chemically it derives from podophyllotoxin, a toxin found in the American Mayapple (Sanford *et al.*, 1990; Ernst & Derendorf, 1995). Design, synthesis, and biological evaluation of novel etoposide analogs bearing pyrrolecarboxamidino group as DNA topoisomerase II inhibitors have been reported (Ji *et al.*, 1997). Two 4'-propylcarbonoxy derivatives of etoposide were synthesized and evaluated as potential prodrugs for anticancer therapy (Wrasidlo *et al.*, 2002). Structures of few derivatives of etoposide are published, viz, 10-hydroxy-1-oxoeremophila-7(11),8(9)-dien-12,8-olide (Wu *et al.*, 2005), (5*R*,5aR,8aR,9*S*)-5-(3,4-dihydroxy-5-methoxyphenyl) -9-fluoro-5,8,8a,9-tetrahydrofuro[3',4':6,7]naphtho[2,3-d] -1,3-dioxol-6(5aH)-one acetone solvate (Zhou *et al.*, 2005), (5aR,8aR,9*R*)-9-(3,4,5-trimethoxyphenyl)-5a,6,8a,9-tetrahydrofuro[3',4': 6,7]naphtho[2,3-d][1,3]dioxole-5,8-dione (Shi & Wang, 2003). In view of the importance of the title compound, C_20_H_22_Cl_2_O_10_, (I), a crystal structure is reported here.

The asymmetric unit of title compound, C_20_H_22_Cl_2_O_10_, (I), consists of a 6-{[(benzyloxy)carbonyl]oxy}group and two chloroacetate groups bonded to a 2-methylhexahydropyrano[3,2-d][1,3]dioxin group at the carbon 1,2 and 3 positions of the pyrano ring fused to a dioxin ring, respectively (Fig. 1). The fused [1,3]dioxin and 2-methylhexahydropyrano six-membered rings each adopt a slightly distorted normal chair configuration (Cremer & Pople, 1975) with puckering parameters Q, θ and φ of 0.598 (2) & 0.6025 (19) Å, 2.95 (19)° & 2.81 (18)°, and 33 (5)° & 357 (4)%, respectively (Fig. 2). For an ideal chair, θ has a value of 0 or 180°. The keto groups in each chloroacetate group are arranged in an antiparallel fashion (Torsion angles C2/O7/C16/C8 = 2.2 (3)°; C3/O9/C18/O10 = 4.4 (3)°) and nearly perpendicular to the benzene ring, while the keto group in the 6-{[(benzyloxy)carbonyl]oxy}group is somewhat diagonal to and bisecting the benzyl ring (torsion angle C1/O4/C8/O5 = -3.1 (3)°). The dihedral angle between the mean planes of the dioxin and benzene rings is 42.2 (2)°. An extensive array of intermolecular C—H···O hydrogen bonds exists which involves acceptor oxygen atoms from the three carbonyl groups, two oxygen atoms from the pyran*o*-dioxin rings, a keto oxygen atom in the 6-{[(benzyloxy)carbonyl]oxy}group, donor C—H atoms from an sp^2^ hybridized carbon in the benzene ring and sp^3^ hybridized carbon atoms from the dioxin ring, a methyl group and each chloroacetate group (Table 1). In addition, intermolecular C—H···Cg π-ring interactions also occur between C4—H4A and C14—H14A atoms of the dioxin and benzene rings and a nearby benzene ring (C4—H4A···Cg3 = 3.879 (2) Å (2-x, 1/2+y, 1/2- z) and C14—H14A···Cg3 = 3.818 (4) Å (-1/2+x, -1/2-y, -z), where Cg3 = ring centroid for C10—C15), respectively. Bond lengths and angles are all within expected ranges (Allen *et al.* 1987).

After a geometry optimized MOPAC PM3 computational calculation (Schmidt & Polik 2007) on (I), in vacuo, the dihedral angle between the mean planes of the dioxin and benzene rings became 66.64°, an increase of 24.42°. These observations support a suggestion that a collection of weak intermolecular forces influence the molecular conformation in the crystal and contribute to the packing of these molecules into chains propagating along the [011].

### Experimental

The title compound was obtained as a gift sample from CAD Pharma, Bangalore, India. Suitable crystals were grown from methanol by slow evaporation (m.p.: 385-388 K).

### Refinement

All of the H atoms were placed in their calculated positions and then refined using the riding model with C—H = 0.95-1.00 Å, and with U_iso_(H) = 1.18-1.49U_eq_(C).

### Crystal data

**Table:**

C_20_H_22_Cl_2_O_10_	*F*(000) = 1024
*M**_r_* = 493.28	*D*_x_ = 1.388 Mg m^−^^3^
Orthorhombic, *P*2_1_2_1_2_1_	Mo *K*α radiation, λ = 0.71073 Å
Hall symbol: P 2ac 2ab	Cell parameters from 8966 reflections
*a* = 8.1780 (1) Å	θ = 4.8–32.5°
*b* = 14.9165 (3) Å	µ = 0.33 mm^−^^1^
*c* = 19.3555 (4) Å	*T* = 200 K
*V* = 2361.12 (7) Å^3^	Prism, colorless
*Z* = 4	0.44 × 0.34 × 0.27 mm

### Data collection

**Table:**

Oxford Diffraction Gemini diffractometer	5818 independent reflections
Radiation source: Enhance (Mo) X-ray Source	3677 reflections with *I* > 2σ(*I*)
graphite	*R*_int_ = 0.049
Detector resolution: 10.5081 pixels mm^-1^	θ_max_ = 28.3°, θ_min_ = 4.9°
ω scans	*h* = −10→10
Absorption correction: multi-scan (*CrysAlis RED*; Oxford Diffraction, 2007)	*k* = −19→19
*T*_min_ = 0.821, *T*_max_ = 1.000	*l* = −25→25
30676 measured reflections	

### Refinement

**Table:**

Refinement on *F*^2^	Secondary atom site location: difference Fourier map
Least-squares matrix: full	Hydrogen site location: inferred from neighbouring sites
*R*[*F*^2^ > 2σ(*F*^2^)] = 0.040	H-atom parameters constrained
*wR*(*F*^2^) = 0.084	*w* = 1/[σ^2^(*F*_o_^2^) + (0.0439*P*)^2^] where *P* = (*F*_o_^2^ + 2*F*_c_^2^)/3
*S* = 0.92	(Δ/σ)_max_ < 0.001
5818 reflections	Δρ_max_ = 0.34 e Å^−^^3^
290 parameters	Δρ_min_ = −0.23 e Å^−^^3^
0 restraints	Absolute structure: Flack (1983), 2513 Friedel pairs
Primary atom site location: structure-invariant direct methods	Flack parameter: 0.05 (5)

### Special details

**Table:**

Geometry. All esds (except the esd in the dihedral angle between two l.s. planes) are estimated using the full covariance matrix. The cell esds are taken into account individually in the estimation of esds in distances, angles and torsion angles; correlations between esds in cell parameters are only used when they are defined by crystal symmetry. An approximate (isotropic) treatment of cell esds is used for estimating esds involving l.s. planes.
Refinement. Refinement of *F*^2^ against ALL reflections. The weighted *R*-factor *wR* and goodness of fit *S* are based on *F*^2^, conventional *R*-factors *R* are based on *F*, with *F* set to zero for negative *F*^2^. The threshold expression of *F*^2^ > σ(*F*^2^) is used only for calculating *R*-factors(gt) *etc*. and is not relevant to the choice of reflections for refinement. *R*-factors based on *F*^2^ are statistically about twice as large as those based on *F*, and *R*- factors based on ALL data will be even larger.

### Fractional atomic coordinates and isotropic or equivalent isotropic displacement parameters (Å^2^)

**Table:**

	*x*	*y*	*z*	*U*_iso_*/*U*_eq_	
Cl1	0.46237 (7)	0.35551 (4)	0.03846 (3)	0.05778 (17)	
Cl2	0.51793 (9)	0.59375 (5)	0.14719 (4)	0.0793 (2)	
O1	1.17773 (16)	0.47514 (9)	0.26910 (8)	0.0450 (4)	
O2	1.42110 (16)	0.41973 (10)	0.31523 (8)	0.0520 (4)	
O3	1.21358 (17)	0.23957 (9)	0.22343 (7)	0.0377 (3)	
O4	1.06875 (15)	0.14810 (9)	0.15336 (7)	0.0371 (3)	
O5	1.29642 (18)	0.12683 (10)	0.08848 (8)	0.0456 (4)	
O6	1.11749 (18)	0.01657 (9)	0.11327 (8)	0.0452 (4)	
O7	0.86643 (16)	0.28806 (9)	0.11292 (7)	0.0376 (3)	
O8	0.63005 (18)	0.29606 (12)	0.17188 (8)	0.0542 (4)	
O9	0.86234 (16)	0.43867 (9)	0.21585 (7)	0.0366 (3)	
O10	0.8181 (2)	0.49330 (10)	0.10897 (8)	0.0553 (4)	
C1	1.1148 (2)	0.23894 (13)	0.16396 (11)	0.0346 (5)	
H1A	1.1759	0.2628	0.1232	0.042*	
C2	0.9602 (2)	0.29229 (13)	0.17650 (10)	0.0340 (4)	
H2A	0.8964	0.2650	0.2152	0.041*	
C3	1.0049 (2)	0.38910 (13)	0.19405 (10)	0.0354 (5)	
H3A	1.0567	0.4189	0.1533	0.043*	
C4	1.1217 (2)	0.38731 (13)	0.25368 (11)	0.0349 (5)	
H4A	1.0646	0.3623	0.2951	0.042*	
C5	1.2792 (3)	0.47197 (16)	0.32884 (14)	0.0512 (6)	
H5A	1.2172	0.4459	0.3686	0.061*	
C6	1.3804 (3)	0.32825 (15)	0.29908 (12)	0.0458 (6)	
H6A	1.3251	0.2997	0.3389	0.055*	
H6B	1.4808	0.2938	0.2885	0.055*	
C7	1.2681 (2)	0.32912 (13)	0.23705 (11)	0.0358 (5)	
H7A	1.3271	0.3535	0.1959	0.043*	
C8	1.1749 (3)	0.09907 (14)	0.11513 (11)	0.0367 (5)	
C9	1.2170 (3)	−0.04574 (16)	0.07276 (15)	0.0623 (7)	
H9A	1.3284	−0.0502	0.0924	0.075*	
H9B	1.2256	−0.0250	0.0243	0.075*	
C10	1.1332 (3)	−0.13444 (14)	0.07586 (11)	0.0418 (5)	
C11	1.1874 (3)	−0.20073 (18)	0.12047 (13)	0.0600 (7)	
H11A	1.2783	−0.1907	0.1500	0.072*	
C12	1.1032 (5)	−0.2844 (2)	0.12081 (18)	0.0876 (11)	
H12A	1.1381	−0.3321	0.1497	0.105*	
C13	0.9681 (5)	−0.2943 (2)	0.0776 (2)	0.0910 (10)	
H13A	0.9089	−0.3490	0.0780	0.109*	
C14	0.9205 (5)	−0.2287 (3)	0.03579 (19)	0.0983 (11)	
H14A	0.8289	−0.2372	0.0063	0.118*	
C15	1.0007 (3)	−0.1510 (2)	0.03498 (14)	0.0686 (7)	
H15A	0.9640	−0.1052	0.0046	0.082*	
C16	0.7027 (3)	0.28930 (13)	0.11876 (11)	0.0383 (5)	
C17	0.6253 (3)	0.27921 (16)	0.04854 (12)	0.0501 (6)	
H17A	0.5842	0.2172	0.0430	0.060*	
H17B	0.7084	0.2901	0.0123	0.060*	
C18	0.7850 (3)	0.48932 (14)	0.16859 (13)	0.0395 (5)	
C19	0.6518 (3)	0.54010 (16)	0.20523 (12)	0.0494 (6)	
H19A	0.7019	0.5856	0.2359	0.059*	
H19B	0.5886	0.4981	0.2345	0.059*	
C20	1.3316 (3)	0.56632 (18)	0.34550 (18)	0.0757 (9)	
H20A	1.4078	0.5654	0.3846	0.114*	
H20B	1.2354	0.6022	0.3576	0.114*	
H20C	1.3856	0.5927	0.3052	0.114*	

### Atomic displacement parameters (Å^2^)

**Table:**

	*U*^11^	*U*^22^	*U*^33^	*U*^12^	*U*^13^	*U*^23^
Cl1	0.0480 (3)	0.0624 (4)	0.0629 (4)	0.0086 (3)	−0.0107 (3)	0.0046 (3)
Cl2	0.0861 (5)	0.0687 (5)	0.0832 (5)	0.0365 (4)	−0.0317 (4)	−0.0064 (4)
O1	0.0382 (8)	0.0321 (8)	0.0646 (10)	0.0006 (7)	0.0006 (7)	−0.0154 (7)
O2	0.0352 (8)	0.0465 (10)	0.0741 (11)	0.0012 (7)	−0.0012 (8)	−0.0234 (8)
O3	0.0399 (7)	0.0291 (8)	0.0441 (8)	0.0021 (6)	−0.0029 (7)	−0.0041 (6)
O4	0.0384 (7)	0.0287 (8)	0.0441 (8)	0.0000 (6)	0.0061 (6)	−0.0039 (6)
O5	0.0382 (8)	0.0352 (8)	0.0635 (10)	−0.0013 (7)	0.0091 (7)	−0.0066 (7)
O6	0.0512 (8)	0.0281 (8)	0.0564 (9)	−0.0049 (7)	0.0165 (8)	−0.0094 (7)
O7	0.0386 (8)	0.0397 (9)	0.0344 (8)	0.0017 (6)	0.0018 (7)	−0.0024 (7)
O8	0.0422 (8)	0.0779 (12)	0.0426 (10)	0.0057 (8)	0.0049 (8)	−0.0066 (8)
O9	0.0376 (7)	0.0325 (8)	0.0398 (8)	0.0086 (6)	0.0007 (7)	−0.0017 (6)
O10	0.0732 (11)	0.0471 (10)	0.0457 (10)	0.0091 (9)	0.0007 (9)	0.0060 (8)
C1	0.0397 (11)	0.0251 (11)	0.0392 (12)	−0.0026 (9)	0.0019 (9)	−0.0029 (9)
C2	0.0346 (10)	0.0354 (11)	0.0321 (11)	0.0014 (9)	0.0026 (9)	0.0019 (9)
C3	0.0356 (11)	0.0313 (11)	0.0394 (12)	0.0004 (9)	0.0088 (9)	−0.0015 (9)
C4	0.0332 (10)	0.0308 (12)	0.0406 (12)	−0.0038 (8)	0.0047 (9)	−0.0039 (9)
C5	0.0352 (11)	0.0495 (14)	0.0688 (16)	0.0033 (10)	−0.0041 (12)	−0.0253 (12)
C6	0.0400 (11)	0.0456 (14)	0.0519 (14)	0.0027 (10)	0.0004 (11)	−0.0110 (11)
C7	0.0355 (10)	0.0285 (11)	0.0434 (13)	−0.0010 (9)	0.0040 (9)	−0.0079 (9)
C8	0.0413 (12)	0.0301 (12)	0.0388 (12)	0.0017 (10)	−0.0051 (10)	−0.0039 (10)
C9	0.0675 (15)	0.0362 (14)	0.0832 (18)	−0.0035 (12)	0.0294 (15)	−0.0192 (13)
C10	0.0482 (12)	0.0330 (12)	0.0441 (12)	0.0022 (10)	0.0056 (11)	−0.0114 (11)
C11	0.0559 (14)	0.0621 (19)	0.0620 (16)	0.0183 (14)	0.0053 (13)	−0.0025 (14)
C12	0.119 (3)	0.0480 (19)	0.096 (3)	0.0332 (19)	0.049 (2)	0.0267 (17)
C13	0.109 (3)	0.051 (2)	0.113 (3)	−0.030 (2)	0.028 (3)	−0.024 (2)
C14	0.119 (3)	0.087 (3)	0.089 (2)	−0.035 (2)	−0.008 (2)	−0.025 (2)
C15	0.0832 (19)	0.0692 (19)	0.0534 (16)	−0.0104 (16)	−0.0070 (15)	−0.0092 (14)
C16	0.0428 (12)	0.0312 (12)	0.0409 (13)	0.0055 (10)	−0.0012 (11)	−0.0014 (10)
C17	0.0548 (13)	0.0468 (14)	0.0489 (14)	0.0091 (11)	−0.0107 (12)	−0.0084 (11)
C18	0.0466 (12)	0.0274 (11)	0.0446 (14)	−0.0023 (10)	−0.0083 (11)	−0.0007 (10)
C19	0.0518 (13)	0.0403 (13)	0.0562 (14)	0.0130 (11)	−0.0099 (12)	−0.0024 (11)
C20	0.0435 (13)	0.0595 (18)	0.124 (3)	0.0071 (13)	−0.0108 (15)	−0.0508 (17)

### Geometric parameters (Å, °)

**Table:**

Cl1—C17	1.763 (2)	C5—H5A	1.0000
Cl2—C19	1.761 (2)	C6—C7	1.512 (3)
O1—C4	1.420 (2)	C6—H6A	0.9900
O1—C5	1.424 (3)	C6—H6B	0.9900
O2—C5	1.423 (2)	C7—H7A	1.0000
O2—C6	1.439 (3)	C9—C10	1.491 (3)
O3—C1	1.406 (2)	C9—H9A	0.9900
O3—C7	1.433 (2)	C9—H9B	0.9900
O4—C8	1.355 (2)	C10—C15	1.365 (3)
O4—C1	1.421 (2)	C10—C11	1.386 (3)
O5—C8	1.194 (2)	C11—C12	1.426 (4)
O6—C8	1.318 (2)	C11—H11A	0.9500
O6—C9	1.463 (3)	C12—C13	1.393 (5)
O7—C16	1.343 (2)	C12—H12A	0.9500
O7—C2	1.452 (2)	C13—C14	1.328 (5)
O8—C16	1.192 (2)	C13—H13A	0.9500
O9—C18	1.345 (3)	C14—C15	1.331 (4)
O9—C3	1.444 (2)	C14—H14A	0.9500
O10—C18	1.187 (3)	C15—H15A	0.9500
C1—C2	1.513 (3)	C16—C17	1.507 (3)
C1—H1A	1.0000	C17—H17A	0.9900
C2—C3	1.528 (3)	C17—H17B	0.9900
C2—H2A	1.0000	C18—C19	1.504 (3)
C3—C4	1.499 (3)	C19—H19A	0.9900
C3—H3A	1.0000	C19—H19B	0.9900
C4—C7	1.513 (3)	C20—H20A	0.9800
C4—H4A	1.0000	C20—H20B	0.9800
C5—C20	1.506 (3)	C20—H20C	0.9800
			
C4—O1—C5	109.16 (16)	O5—C8—O4	125.60 (19)
C5—O2—C6	111.76 (15)	O6—C8—O4	106.92 (17)
C1—O3—C7	109.60 (15)	O6—C9—C10	106.67 (18)
C8—O4—C1	115.07 (15)	O6—C9—H9A	110.4
C8—O6—C9	114.21 (17)	C10—C9—H9A	110.4
C16—O7—C2	117.04 (15)	O6—C9—H9B	110.4
C18—O9—C3	117.98 (16)	C10—C9—H9B	110.4
O3—C1—O4	106.07 (15)	H9A—C9—H9B	108.6
O3—C1—C2	110.18 (15)	C15—C10—C11	119.1 (2)
O4—C1—C2	107.64 (14)	C15—C10—C9	120.2 (2)
O3—C1—H1A	110.9	C11—C10—C9	120.8 (2)
O4—C1—H1A	110.9	C10—C11—C12	118.2 (3)
C2—C1—H1A	110.9	C10—C11—H11A	120.9
O7—C2—C1	106.42 (15)	C12—C11—H11A	120.9
O7—C2—C3	110.85 (15)	C13—C12—C11	118.2 (3)
C1—C2—C3	109.45 (15)	C13—C12—H12A	120.9
O7—C2—H2A	110.0	C11—C12—H12A	120.9
C1—C2—H2A	110.0	C14—C13—C12	121.4 (3)
C3—C2—H2A	110.0	C14—C13—H13A	119.3
O9—C3—C4	107.40 (14)	C12—C13—H13A	119.3
O9—C3—C2	110.87 (15)	C13—C14—C15	120.3 (4)
C4—C3—C2	107.87 (15)	C13—C14—H14A	119.9
O9—C3—H3A	110.2	C15—C14—H14A	119.9
C4—C3—H3A	110.2	C14—C15—C10	122.8 (3)
C2—C3—H3A	110.2	C14—C15—H15A	118.6
O1—C4—C3	110.57 (16)	C10—C15—H15A	118.6
O1—C4—C7	108.61 (15)	O8—C16—O7	124.80 (19)
C3—C4—C7	110.53 (16)	O8—C16—C17	125.23 (19)
O1—C4—H4A	109.0	O7—C16—C17	109.96 (19)
C3—C4—H4A	109.0	C16—C17—Cl1	110.66 (16)
C7—C4—H4A	109.0	C16—C17—H17A	109.5
O2—C5—O1	110.07 (17)	Cl1—C17—H17A	109.5
O2—C5—C20	108.63 (18)	C16—C17—H17B	109.5
O1—C5—C20	108.0 (2)	Cl1—C17—H17B	109.5
O2—C5—H5A	110.0	H17A—C17—H17B	108.1
O1—C5—H5A	110.0	O10—C18—O9	125.6 (2)
C20—C5—H5A	110.0	O10—C18—C19	126.8 (2)
O2—C6—C7	107.76 (18)	O9—C18—C19	107.63 (19)
O2—C6—H6A	110.2	C18—C19—Cl2	112.21 (17)
C7—C6—H6A	110.2	C18—C19—H19A	109.2
O2—C6—H6B	110.2	Cl2—C19—H19A	109.2
C7—C6—H6B	110.2	C18—C19—H19B	109.2
H6A—C6—H6B	108.5	Cl2—C19—H19B	109.2
O3—C7—C6	109.09 (17)	H19A—C19—H19B	107.9
O3—C7—C4	109.16 (15)	C5—C20—H20A	109.5
C6—C7—C4	108.45 (17)	C5—C20—H20B	109.5
O3—C7—H7A	110.0	H20A—C20—H20B	109.5
C6—C7—H7A	110.0	C5—C20—H20C	109.5
C4—C7—H7A	110.0	H20A—C20—H20C	109.5
O5—C8—O6	127.48 (19)	H20B—C20—H20C	109.5
			
C7—O3—C1—O4	178.93 (14)	O2—C6—C7—O3	−174.90 (16)
C7—O3—C1—C2	−64.85 (19)	O2—C6—C7—C4	−56.1 (2)
C8—O4—C1—O3	−87.73 (18)	O1—C4—C7—O3	178.26 (15)
C8—O4—C1—C2	154.34 (16)	C3—C4—C7—O3	−60.3 (2)
C16—O7—C2—C1	148.00 (16)	O1—C4—C7—C6	59.5 (2)
C16—O7—C2—C3	−93.07 (19)	C3—C4—C7—C6	−179.03 (16)
O3—C1—C2—O7	179.99 (14)	C9—O6—C8—O5	−0.7 (3)
O4—C1—C2—O7	−64.77 (19)	C9—O6—C8—O4	179.11 (18)
O3—C1—C2—C3	60.1 (2)	C1—O4—C8—O5	−3.1 (3)
O4—C1—C2—C3	175.39 (14)	C1—O4—C8—O6	177.14 (15)
C18—O9—C3—C4	145.41 (17)	C8—O6—C9—C10	−179.41 (19)
C18—O9—C3—C2	−96.97 (19)	O6—C9—C10—C15	78.9 (3)
O7—C2—C3—O9	71.06 (18)	O6—C9—C10—C11	−100.5 (2)
C1—C2—C3—O9	−171.86 (16)	C15—C10—C11—C12	1.0 (3)
O7—C2—C3—C4	−171.62 (14)	C9—C10—C11—C12	−179.6 (2)
C1—C2—C3—C4	−54.5 (2)	C10—C11—C12—C13	−1.7 (4)
C5—O1—C4—C3	175.95 (16)	C11—C12—C13—C14	1.6 (5)
C5—O1—C4—C7	−62.6 (2)	C12—C13—C14—C15	−0.8 (6)
O9—C3—C4—O1	−64.92 (19)	C13—C14—C15—C10	0.1 (5)
C2—C3—C4—O1	175.53 (15)	C11—C10—C15—C14	−0.2 (4)
O9—C3—C4—C7	174.79 (15)	C9—C10—C15—C14	−179.6 (3)
C2—C3—C4—C7	55.2 (2)	C2—O7—C16—O8	2.2 (3)
C6—O2—C5—O1	−62.2 (2)	C2—O7—C16—C17	−176.82 (16)
C6—O2—C5—C20	179.8 (2)	O8—C16—C17—Cl1	45.1 (3)
C4—O1—C5—O2	63.6 (2)	O7—C16—C17—Cl1	−135.95 (16)
C4—O1—C5—C20	−177.92 (18)	C3—O9—C18—O10	4.4 (3)
C5—O2—C6—C7	58.2 (2)	C3—O9—C18—C19	−174.89 (16)
C1—O3—C7—C6	−177.39 (16)	O10—C18—C19—Cl2	11.2 (3)
C1—O3—C7—C4	64.27 (19)	O9—C18—C19—Cl2	−169.47 (15)

### Hydrogen-bond geometry (Å, °)

**Table:**

Cg3 is the centroid of the C10—C15 ring.

**Table:**

*D*—H···*A*	*D*—H	H···*A*	*D*···*A*	*D*—H···*A*
C6—H6B···O8^i^	0.99	2.57	3.235 (3)	125
C13—H13A···O10^ii^	0.95	2.54	3.452 (4)	162
C17—H17B···O5^iii^	0.99	2.42	3.310 (3)	149
C19—H19A···O3^iv^	0.99	2.52	3.460 (3)	158
C19—H19B···O2^v^	0.99	2.38	3.364 (3)	170
C20—H20B···O4^iv^	0.98	2.59	3.494 (3)	154
C4—H4A···Cg3^iv^	1.00	2.89	3.879 (2)	171
C14—H14A···Cg3^vi^	0.95	2.87	3.818 (4)	173

Symmetry codes: (i) *x*+1, *y*, *z*; (ii) *x*, *y*−1, *z*; (iii) *x*−1/2, −*y*+1/2, −*z*; (iv) −*x*+2, *y*+1/2, −*z*+1/2; (v) *x*−1, *y*, *z*; (vi) *x*−1/2, −*y*−1/2, −*z*.

## References

[bb1] Allen, F. H., Kennard, O., Watson, D. G., Brammer, L., Orpen, A. G. & Taylor, R. (1987). *J. Chem. Soc. Perkin Trans. 2*, pp. S1–19.

[bb2] Budavari, S. (1989). Editor. *The Merck Index: An Encyclopedia of Chemicals, Drugs and Biologicals*, Rahway, NJ: Merck & Co.

[bb3] Cremer, D. & Pople, J. A. (1975). *J. Am. Chem. Soc.* **97** 1354–1355.

[bb4] Ernst, M. & Derendorf, H. (1995). *Drug Actions: Basic Principles and Therapeutic Aspects* Stuttgart: Medpharm Scientific Publishers.

[bb5] Flack, H. D. (1983). *Acta Cryst.* A**39**, 876–881.

[bb6] Ji, Z., Wang, H.-K., Bastow, K. F., Zhu, X.-K., Cho, S. J., Cheng, Y.-C. & Lee, K.-H. (1997). *Bioorg. Med. Chem. Lett.* **7**, 607–612.

[bb7] Oxford Diffraction (2007). *CrysAlis PRO* and *CrysAlis RED* Oxford Diffraction Ltd, Abingdon, England.

[bb8] Sanford, G. L., Gilman, A. & Gilman, A. G. (1990). *The Pharmacological Basis of Therapeutics*, 8th Edition. Elmsford, NY, Pergamon Press.

[bb9] Schmidt, J. R. & Polik, W. F. (2007). *WebMO Pro* WebMO, LLC: Holland, MI, USA, available from http://www.webmo.net.

[bb10] Sheldrick, G. M. (2008). *Acta Cryst.* A**64**, 112–122. 10.1107/S0108767307043930 18156677

[bb11] Shi, J.-F. & Wang, Y.-G. (2003). *Acta Cryst.* E**59**, o756–o758.

[bb12] Wrasidlo, W., Schröder, U., Bernt, K., Hübener, N., Shabat, D., Gaedicke, G. & Lode, H. (2002). *Bioorg. Med. Chem. Lett.* **12**, 557–560. 10.1016/s0960-894x(01)00801-0 11844671

[bb13] Wu, Q.-X., Shi, Y.-P. & Shi, Y.-P. (2005). *Acta Cryst.* E**61**, o3502–o3504.

[bb14] Zhou, F.-Y., Jian, S.-Z., He, X.-Y. & Wang, Y.-G. (2005). *Acta Cryst.* E**61**, o1374–o1376.

